# Custo-Efetividade da Oxigenação por Membrana Extracorpórea Venoarterial no Choque Cardiogênico Refratário: Um Estudo na Perspectiva Brasileira

**DOI:** 10.36660/abc.20230672

**Published:** 2024-07-25

**Authors:** Sérgio Renato da Rosa Decker, Rodrigo Vugman Wainstein, Fernando Luis Scolari, Priscila Raupp da Rosa, Daniel Schneider, Debora Vacaro Fogazzi, Geraldine Trott, Jonas Wolf, Cassiano Teixeira, Marciane Maria Rover, Luiz Antônio Nasi, Luis Eduardo Rohde, Carisi Anne Polanczyk, Regis Goulart Rosa, Eduardo Gehling Bertoldi

**Affiliations:** 1 Serviço de Medicina Interna Hospital Moinhos de Vento Porto Alegre RS Brasil Serviço de Medicina Interna, Hospital Moinhos de Vento, Porto Alegre, RS – Brasil; 2 PROADI SUS Hospital Moinhos de Vento Porto Alegre RS Brasil Escritório de Pesquisa PROADI-SUS, Hospital Moinhos de Vento, Porto Alegre, RS – Brasil; 3 Programa de Pós Graduação em Cardiologia e Ciências Cardiovasculares Universidade Federal do Rio Grande do Sul Porto Alegre RS Brasil Programa de Pós Graduação em Cardiologia e Ciências Cardiovasculares - Universidade Federal do Rio Grande do Sul, Porto Alegre, RS – Brasil; 4 Serviço de Cardiologia Hospital Moinhos de Vento Porto Alegre RS Brasil Serviço de Cardiologia do Hospital Moinhos de Vento, Porto Alegre, RS – Brasil; 5 Serviço de Cardiologia, Hospital de Clinicas de Porto Alegre Porto Alegre RS Brasil Serviço de Cardiologia, Hospital de Clinicas de Porto Alegre, Porto Alegre, RS – Brasil; 6 Faculdade de Medicina Universidade Nove de Julho São Paulo SP Brasil Faculdade de Medicina, Universidade Nove de Julho, São Paulo, SP – Brasil; 7 Escritório de Gestão da Prática Clínica Hospital Moinhos de Vento Porto Alegre RS Brasil Escritório de Gestão da Prática Clínica, Hospital Moinhos de Vento, Porto Alegre, RS – Brasil; 8 Faculdade de Medicina Universidade Federal de Pelotas Pelotas RS Brasil Faculdade de Medicina, Universidade Federal de Pelotas, Pelotas, RS – Brasil

**Keywords:** Oxigenação por Membrana Extracorpórea, Choque Cardiogênico, Custos e Análise de Custo

## Abstract

**Fundamento:**

O choque cardiogênico (CC) refratário está associado com altas taxas de mortalidade, e o uso de oxigenação por membrana extracorpórea venoarterial (VA-ECMO, do inglês *venoarterial extracorporeal membrane oxygenation* ) como uma opção terapêutica tem gerado discussões. Nesse sentido, sua custo-efetividade, principalmente em países de baixa e média renda como o Brasil, continua incerto.Objetivos: Conduzir uma análise de custo-efetividade na perspectiva do Sistema Único de Saúde (SUS) para avaliar a custo-efetividade de VA-ECMO combinado com o tratamento padrão em comparação ao tratamento padrão isolado em pacientes adultos com CC refratário.

**Métodos:**

Acompanhamos uma coorte de pacientes com CC refratário tratados com VA-ECMO em centros de assistência terciária do sul brasileiro. Coletamos dados de desfechos e custos hospitalares. Realizamos uma revisão sistemática para complementar nossos dados e usamos o modelo de Markov para estimar a razão de custo-efetividade incremental (RCEI) por ano de vida ajustado pela qualidade (QALY) e por ano de vida ganho.

**Resultados:**

Na análise do caso-base, a VA-ECMO gerou uma RCEI de Int$ 37 491 por QALY. Análises de sensibilidade identificaram o custo de internação, o risco relativo de sobrevida, e a sobrevida do grupo submetido à VA-ECMO como principais variáveis influenciando os resultados. A análise de sensibilidade probabilística mostrou um benefício do uso de VA-ECMO, com uma probabilidade de 78% de custo-efetividade no limiar recomendado de disposição a pagar.

**Conclusões:**

Nosso estudo sugere que, dentro do SUS, VA-ECMO pode ser uma terapia custo-efetiva para o CC refratário. Contudo, a escassez de dados sobre a eficácia e de ensaios clínicos recentes que abordem seus benefícios em subgrupos específicos de pacientes destaca a necessidade de mais pesquisas. Ensaios clínicos rigorosos, incluindo perfis diversos de pacientes, são essenciais para confirmar a custo-efetividade com uso de VA-ECMO e assegurar acesso igualitário a intervenções médicas avançadas dentro dos sistemas de saúde, especialmente em países com desigualdades socioeconômicas como o Brasil.

## Introdução

O choque cardiogênico (CC) refratário está associado a um prognóstico ruim, com taxas de mortalidade que variam entre 40 e 88%.^1 , 2^ Coortes contemporâneas têm mostrado melhor sobrevida em centros que atendem grandes volumes de pacientes e integram a Oxigenação por Membrana Extracorpórea Venoarterial (VA-ECMO, do inglês *venoarterial extracorporeal membrane oxygenation* ) para restaurar perfusão tecidual, reduzir lesão de órgão, e estabilizar pacientes com CC refratário, como uma ponte para a recuperação, transplante cardíaco, ou outras decisões terapêuticas.^1 , 3 - 6^ No entanto, ensaios clínicos com pacientes com infarto do miocárdio (IM) não conseguiram demonstrar um benefício clínico claro.^7^

Além disso, dada à necessidade de uma Unidade de Terapia Intensiva (UTI)^4^ especializada e os custos dos equipamentos,^6 - 8^ o processo de tomada de decisão para a incorporação da tecnologia VA-ECMO requer uma avaliação abrangente que inclui análise de custo-utilidade e análise do impacto orçamentário. Essa avaliação é particularmente relevante para os sistemas de saúde em países de renda baixa e média.

Acompanhamos uma coorte de pacientes com CC refratário tratado com VA-ECMO em centros de assistência terciária localizados na região sudeste do Brasil, coletando dados sobre desfechos e custos hospitalares. Realizamos uma análise de custo utilidade, além de uma revisão da literatura, para comparar a efetividade da VA-ECMO combinada com terapia padrão *versus* terapia padrão isolada em pacientes adultos com CC refratário independentemente da etiologia em relação à evidência atual. Este estudo foi conduzido pela perspectiva do Sistema Único de Saúde (SUS).

## Métodos

Como parte de um programa de pesquisa nacional focado na avaliação da viabilidade de se integrar a VA-ECMO no SUS, conduzimos uma análise de custo-utilidade em um estudo prospectivo do tipo coorte de pacientes com CC refratário tratados com VA-ECMO. O estudo foi conduzido em quatro centro de assistência terciária do sudeste brasileiro entre abril de 2017 e dezembro de 2020. Os centros foram incluídos no programa “Qualificação do uso de Dispositivos de Assistência Circulatória no SUS”. A descrição dos dados foi conduzida de acordo com as diretrizes CHEERS.^8^

Para serem considerados elegíveis, os centros precisavam apresentar um laboratório de cateterismo 24/7, uma equipe especializada em insuficiência cardíaca capaz de empregar dispositivos temporários de assistência circulatória mecânica (ACM) e uma equipe de cirurgia cardíaca. Ainda, os centros deviam estar localizados na região sudeste do país. Todos os centros participantes passaram por um treinamento, seguindo as diretrizes estabelecidas pela ELSO ( *Extracorporeal Life Support Organization* ),^9^ incluindo seminários e práticas em cenários de simulação. No entanto, os centros tinham autonomia para implementar estratégias de tratamento do CC com base nos recursos locais, incluindo algoritmos, equipes de CC, uso (obrigatório ou caso a caso) de catéteres de artéria pulmonar, seleção de equipamentos e protocolos de desmame.^1^

Outros dados não disponíveis dessa coorte foram obtidos por meio de uma revisão sistemática da literatura. Buscamos nos bancos de dados PubMed, Cochrane CENTRAL e EMBASE estudos relatando desfechos em pacientes com CC tratados com VA-ECMO, além da busca manual de referências dos artigos encontrados. O método detalhado de nossa revisão está apresentado no material suplementar, e os dados extraídos da literatura e utilizados em nosso modelo estão descritos nas Tabelas 1 e 2.

Os resultados são apresentados como razão custo-efetividade incremental por ano de vida ajustado pela qualidade (QALY, do inglês *quality-adjusted life year* ) e anos de vida ganhos, e comparados com o limiar da disposição a pagar (DAP) recomendado pela Comissão Nacional de Incorporação de Tecnologias (CONITEC) no SUS.^10^

### Pacientes

Os pacientes incluídos no estudo apresentavam CC e tinham idade superior a 18 anos de idade. O CC foi definido como pressão arterial sistólica (PAS) < 90mmHg por mais de 30 minutos ou necessidade de agentes inotrópicos ou vasopressores para manutenção da PAS > 90mHg, ou índice cardíaco < 2,2 L/min/m^2^ recebendo inotrópicos/vasopressores, sinais de falência de órgãos (débito urinário < 0,5mL/kg/h, nível de lactato >2mmol/L, pele úmida, tempo de enchimento capilar >3s), sem melhora apesar do manejo inicial com ressuscitação volêmica e/ou uso de vasopressores e inotrópicos.^2 - 4^

Trinta e cinco pacientes com CC foram incluídos na coorte VA-ECMO. As características basais foram idade mediana de 55 (42-63) anos, 23 (63%) eram do sexo masculino, e as causas de CC foram: IM agudo (n=13, 37%), insuficiência cardíaca aguda descompensada (n=8, 23%), pós-transplante cardíaco (n= 7, 20%), pós-cardiotomia (n=4, 11%), embolismo pulmonar (n=2, 6%), e miocardite (n=1, 3%).^1^ No total, 61% dos pacientes foram a óbito, 76% desenvolveram complicações, sendo as mais comuns sangramento e infecção.^1^

### Modelo

Construímos uma árvore de decisão comparando o tratamento padrão de CC na UTI, ao tratamento padrão combinado com a VA-ECMO. O modelo foi construído usando o Treeage Pro 2020, R2.1 (TreeAge Software, Williamstown, MA, EUA). O modelo computa a probabilidade de eventos adversos influenciada pela estratégia escolhida; tais probabilidades provêm da coorte local de pacientes com CC,^1^ ou de acordo com os eventos adversos mais relevantes identificados na revisão da literatura. Múltiplos eventos adversos podem ocorrer em qualquer combinação, com probabilidades independentes, influenciados pela estratégia aplicada. O modelo considera as combinações de eventos, e fornece a taxa de sobrevivência hospitalar e a proporção de pacientes com incapacidades relacionadas a eventos adversos hospitalares para cada estratégia. Após a alta hospitalar, a sobrevida e o ganho em QALY em longo prazo são determinados por incapacidades, se presentes na alta, sem influência do tratamento inicial. O horizonte de tempo para o estudo foi o tempo de vida.

**Figura 1:**
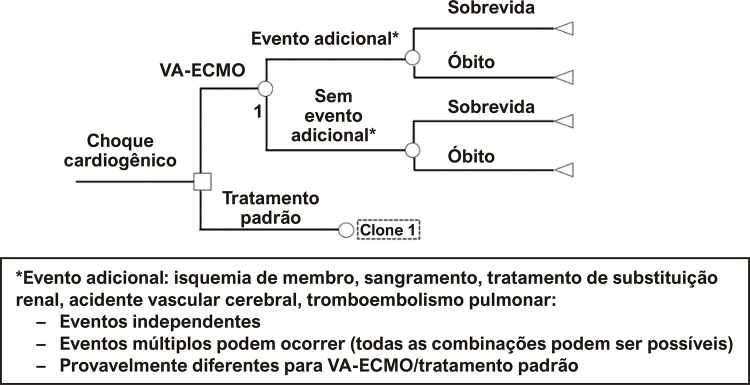
– Representação esquemática do modelo; VA-ECMO: Oxigenação por Membrana Extracorpórea Venoarterial.

Como observado em nossa coorte e nos dados da literatura, uma proporção de pacientes do modelo sobreviverá com uma qualidade de vida equivalente a pacientes com insuficiência cardíaca sintomática, doença cardíaca isquêmica, e um terceiro grupo viverá como indivíduos saudáveis. Detalhes do modelo representado esquematicamente e os dados inseridos são apresentados na Figura 1 e na Tabela 1 .

**Tabela 1:** – Parâmetros inseridos no modelo

Variável	Caso-base	Ref (s)	Análise de sensibilidade	Ref (s)
**Probabilidades**				
AVC – grupo controle	8%	^5^ , §	4% - 16%	^26^ , §
AVC – VA-ECMO	5,9%	c	3% - 12%	^6^ , §
TSR – grupo controle	25%	^27^	12,5% - 50%	§
TSR – VA-ECMO	22%	c	11% - 44%	^27^ ^,^ ^28^ , §
Necessidade de TSR após a alta hospitalar	5%	^29^	3% - 10%	^29^ , §
Isquemia de membros – grupo controle	6%	^27^ , §	3% - 12%	§
Isquemia de membros – VA-ECMO	15%	c	7,3% - 29,4%	c, §
Amputação para isquemia de membros	20%	c	10% - 40%	§
Sangramento – grupo controle	12%	^26^	15% - 60%	§
Sangramento – VA-ECMO	41,2%	c	20,6% - 82,4%	c, §
Anemia após a alta em caso de sangramento	53%	^30^	26,5% - 100%	^30^ , §
Embolia pulmonar – grupo controle	9,1%	^31^	4,5% - 18%	^31^ , §
Embolia pulmonar – VA-ECMO	14,7%	c	7,35% - 29,4%	c, §
HP após embolia pulmonar	3,2%	^32^ ^,^ ^33^	2% - 4,4%	^32^ ^,^ ^33^
Sobrevida – VA-ECMO	54,9%	c, ^6^	41% - 75%	c, ^6^
Sobrevida – grupo controle	30,25%	c, ^6^	23% - 41%	c, ^6^
RR para morte hospitalar – VA-ECMO vs. controle	0,551	c, ^6^	0,375 - 0,809	c, ^6^
IC após a alta	45%	^11^ ^,^ ^12^	40% - 50%	^11^ ^,^ ^12^
DAC após a alta	48%	c	30% - 60%	c, §
**Outras variáveis**				
Sobrevida média – IC (anos)	5,92	^34^	2,52 - 5,95	^35^ ^,^ ^36^
Sobrevida média – DAC (anos)	11,4	^14^ , §	8 - 16	^14^ , §
Sobrevida ajustada – IC (QALY)	4,4	^34^	3,99 - 5,23	^35^ ^,^ ^36^
Sobrevida ajustada – DAC (QALY)	8,46	^14^	6 - 11	^14^
Sobrevida média saudável (anos)	26,5	^15^	20 - 30	^15^ , §
Sobrevida ajustada saudável (QALY)	20,14	^37^ ^,^ ^38^	8,4 - 30	^37^ ^,^ ^38^

AVC: acidente vascular cerebral; TSR: tratamento de substituição renal; HP: hipertensão pulmonar; RR: risco relativo; IC: insuficiência cardíaca; DAC: doença arterial coronariana; c: coorte; §: pressuposto.

### Parâmetros do modelo

Para fornecer dados ao modelo, conduzimos uma revisão sistemática da literatura pelos bancos de dados PubMed, Embase e Cochrane Central em busca de metanálises, estudos intervencionais ou estudos observacionais de VA-ECMO vs. tratamento padrão de CC (material suplementar).

Para a eficácia, dados da coorte local foram combinados com dados de uma metanálise previamente publicada,^6^ encontrada em nossa revisão sistemática. Usamos o programa OpenMeta por metanálise convencional e metanálise de braço único para combinar os resultados. Seguimos dois critérios para integrar nossos achados aos da metanálise.^6^ Primeiro, a metanálise incluiu estudos comparando o grupo VA-ECMO com tratamento padrão. Segundo, a metanálise incorporou os mesmos estudos observacionais identificados em nossa revisão sistemática. Usamos somente estudos incluídos na metanálise^6^ que avaliou a VA-ECMO no CC fora do contexto de parada cardíaca. Assim, a probabilidade de sobrevida hospitalar com VA-ECMO foi calculada (54,9% com VA-ECMO versus 30,25% no grupo controle), bem como o risco relativo da sobrevida hospitalar sem intervenção ( Tabela 1 ). A probabilidade de eventos adversos no grupo VA-ECMO baseou-se nas taxas observadas na coorte local.^1^ A probabilidade de eventos adversos no grupo controle foi obtida de nossa revisão da literatura.

A proporção de pacientes com insuficiência cardíaca e doença cardíaca isquêmica após a alta hospitalar baseou-se nos dados da coorte e dados publicados.^1 , 11 , 12^ O impacto dessas doenças sobre sobrevida e qualidade de vida foi obtido de análises econômicas publicadas anteriormente, que usaram coortes de pacientes de um dos hospitais incluídos neste estudo.^13 , 14^Para pacientes sem comorbidades, a sobrevida foi obtida das tabelas de mortalidade do Instituto Brasileiro de Geografia e Estatística (IBGE).^15^ Valores alternativos foram obtidos da literatura e usados nas análises de sensibilidade. Todas os dados de probabilidade inseridos são apresentados na Tabela 1 . Aplicamos uma taxa de desconto de 5% por ano tanto para a efetividade clínica como para os parâmetros de custos.

### Dados de custo

Para estimar os custos de internação, aplicamos o método de microcusteio para coletar dados de um subgrupo de 11 pacientes de uma coorte local, em três hospitais do Rio Grande do Sul, Brasil. Todos os hospitais incluídos na análise de custo eram hospitais-escola terciários; dois associados ao SUS, e outros ao sistema de saúde suplementar. Os hospitais estavam localizados próximos ao principal escritório da pesquisa, o que facilitou a coleta e a análise dos dados. Todos os custos foram convertidos do Real para Dólar internacional (Int$), usando o último fator de conversão de paridade do poder de compra disponível no site do Banco Mundial, que considerou 1 dólar internacional igual a 2,53 reais (https://data.worldbank.org/indicator/PA.NUS.PPP?locations=BR, acessado em 18 de abril de 2023).

No grupo VA-ECMO, foram considerados os custos relacionados à compra e à implantação, que envolve o custo de aquisição, manutenção periódica, cânulas arteriais, membranas e outros, considerando o número anual esperado de implantes por instituição e o ciclo de vida do equipamento.

Considerando que os dados de microcusteio incluíram gastos relacionados às complicações, o custo médio de internação foi atribuído à coorte inteira de pacientes submetidos à VA-ECMO, e o custo médio de internação para o tratamento padrão foi atribuído ao grupo controle. Assumiu-se que os eventos adversos e as comorbidades no modelo tiveram impacto somente sobre a sobrevida e na qualidade de vida no término do modelo, e não nos custos em longo prazo. Os dados de custos inseridos são apresentados na Tabela 2 .

**Tabela 2:** – Custo e utilidades

Variável	Caso-base	Ref (s)	Análise de sensibilidade	Ref (s)
**Custos**				
Tratamento padrão (Int$)	10.694	mc	5.347 - 22.971	§
VA-ECMO – internação (Int$)	63.060	mc	31.530 - 126.119	§
VA-ECMO – implante (Int$)	12.648	ac	6.324 - 25.297	§
**VA-ECMO - capital**				
*Aquisição (Int$)*	96.933	ac		§
*Tempo de serviço*	10 anos	§	3 – 10 anos	§
*Interesse*	5%	§	3% - 10%	§
*Custos anuais do serviço (Int$)*	2.274	ac	1.137 - 4.547	§
*Implantes per hospital*	5 / ano	c	3 - 10 / ano	§
*Custo por paciente (Int$)*	2.547	c	1.051 - 5.188	c
VA-ECMO – total (Int$)	78.255	calc	38.906 - 156.605	calc.
**Utilidades (decréscimo)**				
AVC (longo prazo)	0,266	^39^ ^,^ ^40^	0,228 - 0,295	^39^ ^,^ ^40^
Amputação (longo prazo)	0,039	^41^	0,023 - 0,059	^41^
HP após embolia pulmonar (longo prazo)	0,70	^33^	0,30 - 0,80	^33^
Anemia (1 ano)	0,052	^41^	0,034 - 0,076	^41^
TSR (longo prazo)	0,571	^41^	0,398 - 0,725	^41^

O custo total com VA-ECMO inclui custos com a internação, implante e por paciente. AVC: acidente vascular cerebral; mc: microcusteio; cr: custo real; c: coorte; calc: calculado de outros parâmetros; HP: hipertensão pulmonar; TSR: tratamento de substituição renal; §: pressuposto.

### Limiar da disposição a pagar

Nós adotamos o limiar oficial de DAP para as condições fatais no SUS: três vezes o produto interno bruto per capita, equivalente a Int$ 54,729 por QALY em 2023.^10 , 16^

### Análise de sensibilidade

Valores alternativos para todos os dados inseridos foram usados para análise de sensibilidade unidirecional. No caso de dados primários obtidos da coorte, os limites para a análise de sensibilidade foram estimados com base no intervalo de valores alternativos identificado na revisão da literatura, ou, no caso de informação não disponível, assumindo-se a metade e o dobro dos valores observados na coorte original.

Nos parâmetros com múltiplos valores encontrados na revisão da literatura, os valores mais altos e os mais baixos foram usados como intervalos para a análise de sensibilidade. Para o risco relativo e estimativas de probabilidade, intervalos de confiança de 95% foram usados como limites na análise de sensibilidade. Para os dados de custo, a metade e o dobro das estimativas basais foram usados como os limites inferiores e superiores da análise de sensibilidade.

Após identificar os parâmetros aos quais o modelo era mais sensível, realizamos análises de sensibilidade bidirecionais, para registrar o efeito da variação simultânea de duas variáveis ao mesmo tempo.

Ainda, realizou-se a análise de sensibilidade probabilística, com variação simultânea de todos os parâmetros. A simulação usou 100 000 ensaios, com distribuições beta para probabilidade e variáveis de utilidade, e distribuições gama para dados de custo e sobrevida.

## Resultados

### Caso base

Na análise principal, o custo médio por paciente do tratamento padrão foi de Int$ 10.694, e o tratamento com VA-ECMO teve um custo médio de Int$ 78.255. Para o horizonte de vida, a sobrevivência média foi de 3,02 anos com o tratamento padrão e 5,49 anos com VA-ECMO; a sobrevivência ajustada pela qualidade de vida mostrou 2,18 QALY para o tratamento padrão e 3,99 QALY para o tratamento com VA-ECMO. Isso resultou em uma razão de custo-efetividade incremental (RCEI) de Int$ 37.491 por QALY. Na análise secundária, o RCEI foi de Int$ 27.432 por ano de vida ganho. A Tabela 3 resume os resultados do caso base.

**Tabela 3:** – Resultados do caso-base

Resultados por QALY			
Estratégia	Custo (Int$)	QALY ganho	RCEI
Tratamento padrão	10.694	2,18	
VA-ECMO	78.255	3,99	37.491 Int$/ QALY
**Resultados por ano de vida**			
**Estratégia**	**Custo (Int$)**	**Anos de vida ganhos**	**RCEI**
Tratamento padrão	10.694	3,02	
VA-ECMO	78.255	5,49	27.432 Int$/ AVG

RCEI: Razão de Custo-Efetividade Incremental; AVG: Anos de vida ganhos.

### Análise de sensibilidade

Análises de sensibilidade unidirecional e bidirecional mostraram que os resultados foram sensíveis principalmente ao custo de internação no grupo VA-ECMO, probabilidade de risco relativo de sobrevida entre os grupos, e sobrevida no grupo VA-ECMO.

**Figura 2:**
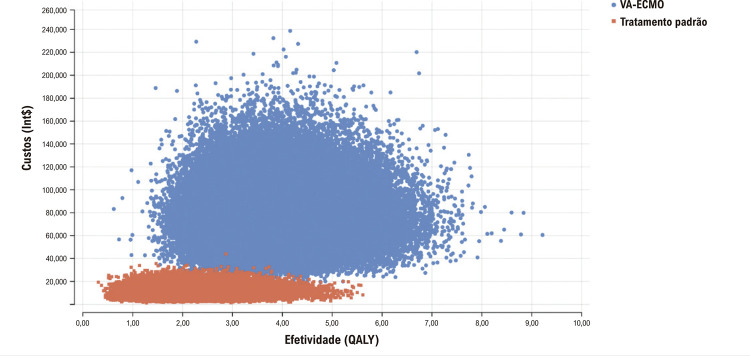
– Dispersão dos resultados de custo e de utilidade da Oxigenação por Membrana Extracorpórea Venoarterial (VA-ECMO) e do tratamento padrão para choque cardiogênico.

**Figura 3:**
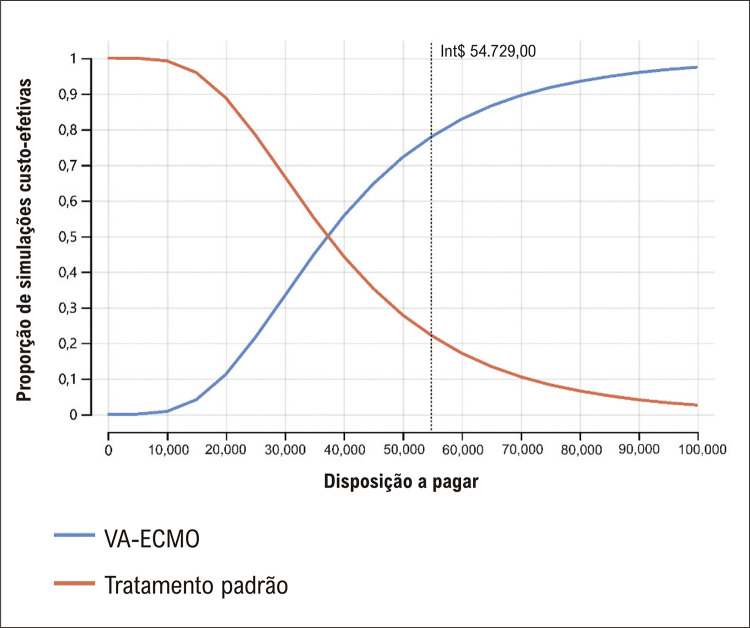
– Curva de aceitabilidade da custo-efetividade (custo por QALY); linha pontilhada representa o limiar da disposição a pagar no Brasil.

Na análise de sensibilidade probabilística com 100 000 ensaios, a estratégia VA-ECMO é consistentemente mais efetiva e mais cara que o tratamento convencional, apesar de uma dispersão relativamente mais ampla dos resultados de custo e utilidade para a VA-ECMO ( Figura 2 ). O gráfico de dispersão da custo-efetividade incremental mostra que 100% das iterações apresentam custo incremental e efetividade positivas. ( Figura Central ). A curva de aceitabilidade de custo-efetividade mostra uma probabilidade de 78% da terapia VA-ECMO ser custo-efetiva no limiar de DAP proposto ( Figura 3 ).

**Figura Central:**
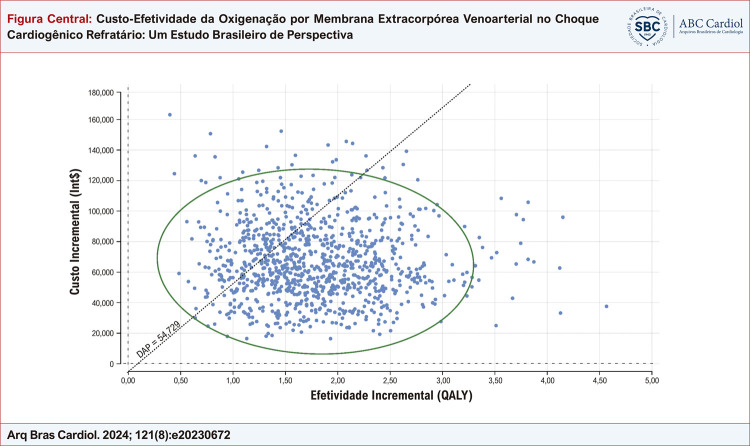
: Custo-Efetividade da Oxigenação por Membrana Extracorpórea Venoarterial no Choque Cardiogênico Refratário: Um Estudo Brasileiro de Perspectiva

Diagrama de dispersão de custo-efetividade incremental (custo por QALY), VA-ECMO vs. Cuidado Padrão. Cada ponto é uma simulação probabilística do modelo, e o círculo verde contém 95% das simulações. A maioria das simulações (78%) situa-se no lado direito da linha do limiar da disposição a pagar, considerada rentável. DAP: Limite de disposição a pagar.

## Discussão

As decisões quanto à implementação de novas tecnologias em saúde de alto custo podem ser desafiadoras para os *stakeholders* e sistemas de saúde, e tentativas de padronização de limiares adequados da DAP estão continuamente evoluindo. Considerando o limiar atual da DAP do SUS, a VA-ECMO parece custo efetiva em nossa principal análise.^10^ Em todo o mundo, a DAP média por QALY é Int$ 34,309;^17^ o limiar da DAP pode ser até três vezes maior para pacientes críticos,^10^ e em alguns países de alta renda, limiares usuais são mais altos,^18^ sugerindo que a VA-ECMO pode também ser custo efetiva para os sistemas de saúde de outros países.

Encontramos poucas evidências econômicas sobre VA-ECMO para adultos. Uma análise dos sistemas de saúde do Canadá e dos Estados Unidos encontrou um gasto de cerca de 18000 dólares canadenses^19 , 20^ e 74500 dólares americanos por paciente, mas não foi quantificado a custo-efetividade. Da perspectiva de um centro de transplante na Finlândia, o custo por paciente tratado com VA-ECMO variou entre 50 000 e 240 000 euros (mediana 130 000 euros) e RCEI da VA-ECMO para CC foi 12 642 euros por QALY ganho.^5^

Nossos dados mostram robustez suficiente, e os principais resultados não foram influenciados por variáveis individuais, exceto aquelas que contêm os elementos chave de custo efetividade da intervenção: custo da VA-ECMO, e sobrevida do paciente. No entanto, apesar das vantagens de nosso estudo usando dados de complicação e custo obtidos localmente – com um método detalhado baseado na técnica de microcusteio e análise de sensibilidade – limitações inerentes ao método e fontes confiáveis insuficientes sobre dados de eficácia devem ser considerados antes da ampla implementação do VA-ECMO. O modelo de Markov requer presunções sobre transição de estados de saúde, e efeitos colaterais que podem não representar exatamente o mundo real.

Recentemente, ensaios randomizados levantaram preocupações válidas sobre o benefício da VA-ECMO em pacientes com IM e CC. No maior ensaio, o ECLS-SHOCK, 420 pacientes com CC causado por IM, com indicação de revascularização, foram aleatoriamente alocados para receberem VA-ECMO ou o tratamento padrão. Os autores excluíram pacientes com mais de 12 horas de CC. Mortalidade por todas as causas em 30 dias não foi diferente entre os grupos (risco relativo 0,98, intervalo de confiança de 95% 0,8 – 1,19; p=0,81), e a ocorrência de sangramento e complicações vasculares periféricas foi maior no grupo VA-ECMO.^21^ No entanto, no ensaio ECLS-SHOCL, 77% dos pacientes foram ressuscitados de parada cardíaca antes da randomização, enquanto nosso foco foi primariamente CC sem parada cardíaca.^5^ Na coorte brasileira, somente 26% dos pacientes sofreram parada cardíaca antes da canulação.^1^ Ainda, ensaios publicados recentemente testando a VA-ECMO para CC incluíram somente pacientes com IM,7 ao passo que na nossa coorte, o IM representou somente 37% dos casos.^1^ O ensaio ECMO-CS, que incluiu pacientes com diferentes etiologias de CC, testou principalmente o tempo de implementação – a VA-ECMO imediata ou não imediata (conservadora). No grupo conservador, 39% requereu suporte com VA-ECMO, o que pode ter diluído o benefício da VA-ECMO quando comparado a de outra abordagem conservadora, na ausência de inclusão a VA-ECMO ao sistema de saúde.^22^

Portanto, nosso estudo destaca que, se comprovada sua eficácia em estudos futuros, o uso de VA-ECMO poderia ser uma opção terapêutica custo-efetiva no contexto SUS. Contudo, é fundamental reconhecer que a desigualdade é um marco dos países de média renda como o Brasil, e a equidade continua uma preocupação premente.^23^ Além disso, manter a resiliência nas UTIs geralmente requer um melhor entendimento de quais pacientes realmente se beneficiam de terapias intensivas.^24^ Assim, antes da ampla implementação de uma terapia de alto custo, ensaios clínicos rigorosos envolvendo um perfil mais diversificado de pacientes com CC, e uma menor incidência de parada cardíaca precedendo o uso do dispositivo são necessários para elucidar o papel de cada doença (além do IM) sobre os desfechos, e avaliar a relação entre os estágios do CC e o benefício da VA-ECMO.^25^ Além disso, o ensaio mencionado difere-se de nossa coorte, em que a maioria dos pacientes apresentavam classificação SCAI ( *the Society for Cardiovascular Angiography and Interventions* ) D (ao contrário de estudos com uma predominância de SCAI C ou E).^1 , 25^ Mais estudos são necessários não só para reduzir incertezas a respeito do custo efetividade dessa terapia, como também orientar médicos e assegurar acesso igualitário às intervenções médicas de ponta dentro do sistema de saúde.

Dada a crescente eficácia da VA-ECMO, outros desafios na sua integração no sistema de saúde brasileiro, da perspectiva dos gestores políticos, incluem o entendimento das implicações orçamentárias da implementação, o estabelecimento de centros adequadamente equipados e treinados para o uso apropriado do equipamento, e o reconhecimento da presença de um efeito da curva de aprendizagem.^1^ Isso destaca a importância de se estabelecer centros especializados em cada região, considerando suas disparidades e expertise, como o caminho ideal de assegurar a implementação efetiva da tecnologia.

## Conclusão

Em resumo, nossa análise de custo utilidade no contexto SUS sugere que a inclusão do VA-ECMO ao tratamento padrão pode oferecer uma opção terapêutica custo-efetiva para pacientes adultos com CC refratário, independentemente da sua causa. Contudo, a escassez de dados robustos de eficácia e ensaios clínicos randomizados recentes que abordaram subgrupos de pacientes destacam a necessidade de mais pesquisas. Ensaios clínicos rigorosos, incluindo um perfil de pacientes mais diversificados e incidência mais baixa de parada cardíaca imediata, são essenciais para confirmar a custo-efetividade do uso de VA-ECMO e assegurar acesso igualitário a intervenções médicas avançadas dentro do sistema de saúde, especialmente em países como o Brasil com diferentes populações de pacientes.
