# Supplementary material for: Custo-Efetividade da Oxigenação por Membrana Extracorpórea Venoarterial no Choque Cardiogênico Refratário: Um Estudo na Perspectiva Brasileira
Source: Arq Bras Cardiol. 2024 Jul 25;121(8):e20230672. [Article in Portuguese] doi: 10.36660/abc.20230672 (PMC11463338; doi:10.36660/abc.20230672)
Supplement: Translation

# Translation: Cost-Utility of Venoarterial Extracorporeal Membrane Oxygenation in Refractory Cardiogenic Shock: A Brazilian Perspective Study

**DOI:** 10.36660/abc.20230672i

**Authors:** Sérgio Renato da Rosa Decker, Rodrigo Vugman Wainstein, Fernando Luis Scolari, Priscila Raupp da Rosa, Daniel Schneider, Debora Vacaro Fogazzi, Geraldine Trott, Jonas Wolf, Cassiano Teixeira, Marciane Maria Rover, Luiz Antônio Nasi, Luis Eduardo Rohde, Carisi Anne Polanczyk, Regis Goulart Rosa, Eduardo Gehling Bertoldi

**Affiliations:** 1 Brazil Serviço de Medicina Interna, Hospital Moinhos de Vento, Porto Alegre, RS – Brazil; 2 Brazil Escritório de Pesquisa PROADI-SUS, Hospital Moinhos de Vento, Porto Alegre, RS – Brazil; 3 Brazil Programa de Pós Graduação em Cardiologia e Ciências Cardiovasculares - Universidade Federal do Rio Grande do Sul, Porto Alegre, RS – Brazil; 4 Brazil Serviço de Cardiologia do Hospital Moinhos de Vento, Porto Alegre, RS – Brazil; 5 Brazil Serviço de Cardiologia, Hospital de Clinicas de Porto Alegre, Porto Alegre, RS – Brazil; 6 Brazil Faculdade de Medicina, Universidade Nove de Julho, São Paulo, SP – Brazil; 7 Brazil Escritório de Gestão da Prática Clínica, Hospital Moinhos de Vento, Porto Alegre, RS – Brazil; 8 Brazil Faculdade de Medicina, Universidade Federal de Pelotas, Pelotas, RS – Brazil

**Keywords:** Extracorporeal Membrane Oxygenation, Cardiogenic Shock, Costs and Cost Analysis

## Abstract

**Background:**

Refractory cardiogenic shock (CS) is associated with high mortality rates, and the use of venoarterial extracorporeal membrane oxygenation (VA-ECMO) as a therapeutic option has generated discussions. Therefore, its cost-effectiveness, especially in low- and middle-income countries like Brazil, remains uncertain.Objectives: To conduct a cost-utility analysis from the Brazilian Unified Health System perspective to assess the cost-effectiveness of VA-ECMO combined with standard care compared to standard care alone in adult refractory CS patients.

**Methods:**

We followed a cohort of refractory CS patients treated with VA-ECMO in tertiary care centers located in Southern Brazilian. We collected data on hospital outcomes and costs. We conducted a systematic review to supplement our data and utilized a Markov model to estimate incremental cost-effectiveness ratios (ICERs) per quality-adjusted life year (QALY) and per life-year gained.

**Results:**

In the base-case analysis, VA-ECMO yielded an ICER of Int$ 37,491 per QALY. Sensitivity analyses identified hospitalization cost, relative risk of survival, and VA-ECMO group survival as key drivers of results. Probabilistic sensitivity analysis favored VA-ECMO, with a 78% probability of cost-effectiveness at the recommended willingness-to-pay threshold.

**Conclusions:**

Our study suggests that, within the Brazilian Health System framework, VA-ECMO may be a cost-effective therapy for refractory CS. However, limited efficacy data and recent trials questioning its benefit in specific patient subsets highlight the need for further research. Rigorous clinical trials, encompassing diverse patient profiles, are essential to confirm cost-effectiveness and ensure equitable access to advanced medical interventions within healthcare systems, particularly in socio-economically diverse countries like Brazil.

**Central Illustration:** : Cost-Utility of Venoarterial Extracorporeal Membrane Oxygenation in Refractory Cardiogenic Shock: A Brazilian Perspective Study

Dispersion diagram of incremental cost-effectiveness (cost per QALY), VA-ECMO vs. Standard Care. Each point is a probabilistic simulation of the model, and the green circle contains 95% of the simulations. Most of the simulations (78%) are on the right side of the willingness-to-pay threshold line, considered cost-effective. WTP: Willingness-to-pay threshold.

## Introduction

Refractory cardiogenic shock (CS) is associated with a poor prognosis with mortality rates ranging from 40% to 88%.^1 , 2^Contemporary cohorts have exhibited enhanced survival within high-volume centers that integrate venoarterial extracorporeal membrane oxygenation (VA-ECMO) to restore tissue perfusion, reduce organ damage, and stabilize patients with refractory CS, as a bridge to recovery, heart transplant, or other treatment decisions.^1 , 3 - 6^However, clinical trials of myocardial infarction (MI) patients have failed to demonstrate a clear clinical benefit.^7^

Moreover, due to the need for a specialized Intensive Care Unit (ICU)^4^and costs of equipments,^6 - 8^the decision-making process for incorporation of VA-ECMO technology requires a comprehensive evaluation through cost-utility and budget-impact analyses. This evaluation is particularly relevant for health systems in low- and middle-income countries.

We followed a cohort of patients treated with VA-ECMO for refractory CS in Southern Brazilian tertiary care centers, collecting data on hospital outcomes and costs. We conducted a cost-utility analysis, supplemented by a literature review, to compare the effectiveness of VA-ECMO combined with standard care versus standard care alone among adult patients with refractory CS regardless of etiology and compare it with current evidence. This study was carried out from the perspective of the Brazilian Unified Health System (SUS).

## Methods

As part of a national research program focused on assessing the viability of integrating VA-ECMO into SUS, we conducted a cost-utility analysis in a prospective cohort study of patients with refractory CS who received VA-ECMO treatment. The study was conducted at four tertiary care centers in Southern Brazil between April 2017 and December 2020. The centers were enrolled in the program “ *Qualification for use of mechanical circulatory support devices in the Brazilian Unified Health System* ”. The reporting was conducted according to CHEERS guidelines.^8^

To be eligible for enrollment, the centers were required to have a 24/7 catheterization laboratory, a specialized heart failure team capable of employing temporary mechanical circulatory support (MCS) devices and a cardiac surgery team. Also, they should be located at southern Brazil. All participating centers underwent training in MCS utilization following the guidelines established by the Extracorporeal Life Support Organization (ELSO),^9^including seminars and hands-on experience with simulated scenarios. However, centers had the autonomy to implement CS treatment strategies based on local resources, including algorithms, dedicated CS teams, mandatory or case-by-case use of pulmonary artery catheters, device selection, and weaning protocols.^1^

Additional input data not available from this cohort were obtained from systematic review of the literature. We searched PubMed, Cochrane CENTRAL and EMBASE for studies reporting outcomes in patients treated with VA-ECMO for CS, with further manual search of references from retrieved manuscripts. The detailed search method of our review is presented in the supplementary appendix, and data extracted from literature and used in our model are described in Tables 1 and 2.

The results are presented as incremental cost-effectiveness ratio per quality-adjusted life year (QALY) and life-year gained and compared with the willingness-to-pay (WTP) threshold recommended by the National Commission for Incorporation of Technologies in the Brazilian Unified Health System (CONITEC).^10^

### Patients

Patients included had refractory CS and were older than 18 years of age. CS was defined as systolic blood pressure (SBP) <90mmHg for more than 30min or the need of inotropes/vasopressors to maintain SBP >90mmHg, or cardiac index <2.2 L/min/m^2^receiving inotropes/vasopressors, signs of end-organ damage (urine output <0.5mL/kg/h, lactate level >2mmol/L, clammy skin, capillary filling time >3s), without improvement despite the initial management with volume resuscitation and/or use of vasopressors/inotropes.^2 - 4^

Thirty-five CS patients were included in the VA-ECMO cohort. The baseline characteristics were a median age of 55 (interquartile range, 42-63) years old, 23 (63%) were male, and the causes for CS were: acute MI 13 (37%), acute decompensated heart failure 8 (23%), post-heart transplant 7 (20%), post-cardiotomy 4 (11%), pulmonary embolism 2 (6%), and myocarditis 1 (3%).^1^Overall, 61% of patients died, 76% experienced complications, with the most common being bleeding and infection.^1^

### Model

We built a decision tree comparing standard care of CS in ICU, to standard care with the addition of VA-ECMO. Model was built using Treeage Pro 2020, R2.1. (TreeAge Software, Williamstown, MA, USA). The model computes the probability of adverse events influenced by the strategy chosen; these probabilities come from the local cohort of CS patients,^1^or according to the most relevant adverse events identified in the literature review. Multiple adverse events can happen in any combination, with independent probabilities, influenced by the allocated strategy. The model considers the event combinations, and outputs the hospital survival rate and the proportion of patients with disabilities related to in-hospital adverse events for each strategy. After hospital discharge, long-term survival and QALY gains are determined by disabilities, if present at discharge, without further influence from initial treatment allocation. The time horizon for the study was life-time.

As observed in our cohort and literature data, a proportion of modeled patients will survive with a quality of life equivalent to patients with symptomatic heart failure, ischemic heart disease, and a third subgroup will live as healthy individuals. Details of schematically represented model and model inputs are shown in Figure 1 , Table 1 and Table 2 .

**Figure 1:** – Schematic representation of the model. VA-ECMO: venoarterial extracorporeal membrane oxygenation.

**Table 1:** – Input parameters for the model

Variable	Base-case	Ref (s)	Sensitivity analysis	Ref (s)
**Probabilities**				
Stroke – control group	8%	^5^ , §	4% - 16%	^26^ , §
Stroke – VA-ECMO	5.9%	c	3% - 12%	^6^ , §
RRT – control group	25%	^27^	12.5% - 50%	§
RRT – VA-ECMO	22%	c	11% - 44%	^27^ ^,^ ^28^ , §
Need for RRT after discharge	5%	^29^	3% - 10%	^29^ , §
Limb ischemia – control group	6%	^27^ , §	3% - 12%	§
Limb ischemia – VA-ECMO	15%	c	7.3% - 29.4%	c, §
Amputation if limb ischemia	20%	c	10% - 40%	§
Bleeding – control group	12%	^26^	15% - 60%	§
Bleeding – VA-ECMO	41.2%	c	20.6% - 82.4%	c, §
Post-discharge anemia if bleeding	53%	^30^	26.5% - 100%	^30^ , §
PE – control group	9.1%	^31^	4.5% - 18%	^31^ , §
PE – VA-ECMO	14.7%	c	7.35% - 29.4%	c, §
PH after PE	3.2%	^32^ ^,^ ^33^	2% - 4.4%	^32^ ^,^ ^33^
Survival – VA-ECMO	54.9%	c, ^6^	41% - 75%	c, ^6^
Survival – control group	30.25%	c, ^6^	23% - 41%	c, ^6^
RR for in-hospital death – VA-ECMO vs. control	0.551	c, ^6^	0.375 - 0.809	c, ^6^
HF after discharge	45%	^11^ ^,^ ^12^	40% - 50%	^11^ ^,^ ^12^
CHD after discharge	48%	c	30% - 60%	c, §
**Other variables**				
Average survival HF (years)	5.92	^34^	2.52 - 5.95	^35^ ^,^ ^36^
Average survival CHD (years)	11.4	^14^ , §	8 - 16	^14^ , §
Adjusted survival HF (QALY)	4.4	^34^	3.99 - 5.23	^35^ ^,^ ^36^
Adjusted survival CHD (QALY)	8.46	^14^	6 - 11	^14^
Average survival healthy (years)	26.5	^15^	20 - 30	^15^ , §
Adjusted survival healthy (QALY)	20.14	^37^ ^,^ ^38^	8.4 - 30	^37^ ^,^ ^38^

RRT: renal replacement therapy; PE: pulmonary embolism; PH: pulmonary hypertension; RR: relative risk; HF: heart failure; CHD: coronary heart disease; c: cohort; §: assumption; QALY: quality-adjusted life year.

**Table 2:** – Cost and Utilities

Variable	Base-case	Ref (s)	Sensitivity analysis	Ref (s)
**Costs**				
Standard care (Int$)	10,694	mc	5,347 - 22,971	§
VA-ECMO – hospitalization (Int$)	63,060	mc	31,530 - 126,119	§
VA-ECMO – implant (Int$)	12,648	ac	6,324 - 25,297	§
VA-ECMO - capital				
*Acquisition (Int$)*	96,933	ac		§
*Service time*	10 years	§	3 – 10 years	§
*Interest*	5%	§	3% - 10%	§
*Annual service costs (Int$)*	2,274	ac	1,137 - 4,547	§
*Implants per hospital*	5 / year	c	3 - 10 / year	§
*Cost per patient (Int$)*	2,547	c	1,051 - 5,188	c
VA-ECMO – total (Int$)	78,255	calc	38,906 - 156,605	calc.
**Utilities (decrement)**				
Stroke (long term)	0.266	^39^ ^,^ ^40^	0.228 - 0.295	^39^ ^,^ ^40^
Amputation (long term)	0.039	^41^	0.023 - 0.059	^41^
PH after PE (long term)	0.70	^33^	0.30 - 0.80	^33^
Anemia (during 1 year)	0.052	^41^	0.034 - 0.076	^41^
RRT (long term)	0.571	^41^	0.398 - 0.725	^41^

Mc: micro-costing; ac: actual cost; c: cohort; calc: calculated from other parameters; PE: pulmonary embolism; PH: pulmonary hypertension; RRT: renal replacement therapy; §: assumption. VA-ECMO total cost includes VA-ECMO hospitalization, implant, and per patient costs.

### Model parameters

To provide supplementary data for inputs, we performed a systematic review of literature, searching PubMed, Embase and Cochrane Central for meta-analyses, interventional studies, or observational studies of VA-ECMO vs. standard treatment of CS (Supplementary Appendix).

For the efficacy input, local cohort data were combined with data from a previously published meta-analysis,^6^found in our systematic review. We used the OpenMeta software by conventional and single-arm meta-analysis to combine the results. We followed two rationales to integrate our findings with the previously published meta-analysis.^6^Firstly, it encompassed studies comparing the VA-ECMO group with standard treatment. Secondly, the meta-analysis incorporated the same observational studies identified in our systematic review. We only used studies included in the published meta-analysis^6^that evaluated VA-ECMO in CS outside cardiac arrest context. Thus, the probability of hospital survival with VA-ECMO was calculated (54.9% with VA-ECMO *versus* 30.25% in control group), as well as the relative risk of hospital survival without intervention ( Table 1 ). Probability of adverse events in the VA-ECMO group was based on observed rates in the local cohort.^1^The probability of adverse events in the control group was obtained from our literature review.

The proportion of patients with heart failure and ischemic heart disease after hospital discharge was based on cohort data and published data.^1 , 11 , 12^The impact of these diseases on survival and quality of life was obtained from previously published economic analyses, which used cohorts of patients from one of the hospitals included in this study.^13 , 14^For patients without comorbidities, survival was obtained from the National Institute of Statistics (IBGE) mortality tables.^15^Alternative values were obtained from the literature and used in sensitivity analyses. All probability inputs are shown in Table 1 . We apply a discount rate of 5% per year for both clinical effectiveness and cost parameters.

### Cost data

To estimate hospitalization costs, micro-costing methodology was applied to collect data from a subgroup of 11 patients from the local cohort, in three hospitals in Rio Grande do Sul, Brazil. All hospitals included in cost analysis were tertiary teaching hospitals; two were linked to the Brazilian Unified Health System, and other linked to the supplementary health system; they were located close to the main research office facilitating data collection and analysis. All costs were converted from Brazilian Real to International Dollars (Int$), using the World Bank’s latest available purchasing power parity conversion factor of 2.53 (https://data.worldbank.org/indicator/PA.NUS.PPP?locations=BR, accessed April 18, 2023).

In the VA-ECMO group, costs related to purchase and implantation were considered, which involves the cost of acquisition, periodic maintenance, arterial cannulas, membranes, and others, considering the expected annual number of implants per institution and the equipment life-cycle.

Considering that the micro-cost data included expenses related to complications, mean cost of hospitalization was attributed for the entire cohort of patients undergoing VA-ECMO, and mean hospitalization cost for standard treatment was attributed for the control group. It was assumed that adverse events and model comorbidities impacted only on survival and quality of life at model termination, not in long-term costs. Costs inputs are presented in Table 2 .

### Willingness-To-Pay Threshold

We adopted the official WTP threshold for life-threatening conditions in the Brazilian public health system: three times Brazilian GDP per capita, equivalent to Int$ 54,729 per QALY in 2023.^10 , 16^

### Sensitivity analysis

Alternative values for all input data were used in the one-way sensitivity analysis. In the case of primary data obtained from the cohort, the limits for the sensitivity analysis were estimated based on the range of alternative values identified in the literature review, or, in the case of unavailable information, assuming half and twice the values observed in the original cohort.

In parameters with multiple values found in the literature review, the highest and lowest values were used as ranges for the sensitivity analysis. For relative risk and probability estimates, 95% confidence intervals were used as boundaries for the sensitivity analysis. For cost data, half and twice the base estimates were used as the lower and upper limits of the sensitivity analysis.

After identifying the parameters to which the model was most sensitive, we performed two-way sensitivity analyses, to document the effect of simultaneous variation of two variables at a time.

In addition, probabilistic sensitivity analysis was performed, with simultaneous variation of all parameters. The simulation used 100,000 trials, with beta distributions for probability and utility variables, and gamma distributions for cost and survival data.

## Results

### Base-Case

In the main analysis, the mean cost per patient of the standard treatment was Int$ 10,694, and treatment with VA-ECMO had a mean cost of Int$ 78,255. For the life-time horizon, mean survival was 3.02 years with standard treatment and 5.49 years with VA-ECMO; quality-adjusted survival showed standard a 2.18 QALY for the standard treatment and 3.99 QALY for the VA-ECMO treatment. This resulted in an incremental cost-effectiveness ratio (ICER) of Int$ 37,491 per QALY. In the secondary analysis, ICER was Int$ 27,432 per life-year gained. Table 3 summarizes base-case results.

**Table 3:** – Base-case results

Results per QALY			
Strategy	Cost (Int$)	QALY gained	ICER
Standard care	10,694	2.18	
VA-ECMO	78,255	3.99	37,491 Int$/ QALY
**Results per Life-year Gained**			
**Strategy**	**Cost (Int$)**	**Life-years gained**	**ICER**
Standard care	10,694	3.02	
VA-ECMO	78,255	5.49	27,432 Int$/ LYG

ICER: incremental cost-effectiveness ratio; LYG: life-year gained; QALY: quality-adjusted life year.

### Sensitivity analysis

One-way and two-way sensitivity analyses have shown that results were sensitive mainly to cost of hospitalization in the VA-ECMO group, relative risk probability of survival between the groups, and survival in the VA-ECMO group.

In probabilistic sensitivity analysis with 100,000 trials, the VA-ECMO strategy is consistently more effective and more costly than conventional treatment, despite a relatively wide dispersion of cost and utility outputs for VA-ECMO ( Figure 2 ). The scatterplot of incremental cost-effectiveness shows that 100% of iterations have positive incremental cost and effectiveness (Central Figure). The cost-effectiveness acceptability curve shows a 78% probability of VA-ECMO therapy being cost-effective at the proposed WTP threshold ( Figure 3 ).

**Figure 2:** – Dispersion of cost and utility outputs of VA-ECMO and standard treatment for cardiogenic shock. VA-ECMO: venoarterial extracorporeal membrane oxygenation, QALY: quality-adjusted life year.

**Figure 3:** – Cost-effectiveness acceptability curve (cost per QALY). Dashed line shows Brazilian willingness-to-pay threshold. VA-ECMO: venoarterial extracorporeal membrane oxygenation.

## Discussion

Decisions regarding implementation of new high-cost health technologies can be challenging for stakeholders and health systems, and attempts at standardization of adequate WTP thresholds are continually evolving; considering Brazil’s Unified Health System current WTP threshold, VA-ECMO appears to be cost-effective in our main analysis.^10^Worldwide, mean WTP per QALY is Int$ 34,309;^17^WTP threshold can be up to three times higher for critical care patients,^10^and in some high-income countries usual thresholds were higher,^18^suggesting VA-ECMO may also be cost-effective for other nations’ health systems.

We found scarce previous economic evidence on VA-ECMO for adults. An analysis of the Canadian and United States health systems found a cost of about 18,000 Canadian dollars^19 , 20^and US$ 74,500 per patient, but cost-effectiveness was not quantified. From the perspective of a transplant center in Finland, the cost per patient treated with VA-ECMO ranged between 50,000 and 240,000 Euros (median 130,000 Euros) and ICER of VA-ECMO for CS was 12,642 Euros per QALY gained.^5^

Our model shows sufficient robustness, and the main results were not influenced by individual variables, except for those that contain the key elements of cost and effectiveness of the intervention: VA-ECMO cost, and patient survival. However, despite advantages of our study, using adverse event probabilities and cost data obtained locally – with a detailed method based on the micro-costing technique and sensitivity analysis – limitations inherent to the method and insufficient reliable sources of efficacy data should be considered before wide implementation of VA-ECMO. Markov model requires assumptions about state transitions, efficacy, and collateral effects that could not represent exactly the real world.

Recently, randomized trials have raised valid concerns regarding the benefit of VA-ECMO in MI patients followed by CS. In the largest trial, ECLS-SHOCK trial, 420 patients with CS due to MI for whom early revascularization was planned, were randomly assigned to receive VA-ECMO or usual treatment. The authors excluded patients with more than 12 hours of CS. Death from any cause at 30 days was not different between the groups (relative risk, 0.98; 95% confidence interval, 0.8 – 1.19; p=0.81), and bleeding and peripheral vascular complications were higher in the VA-ECMO group.^21^However, in ECLS-SHOCK, 77% of patients were resuscitated due to cardiac arrest before randomization, whereas our focus was primarily CS without cardiac arrest.^5^In the Brazilian cohort only 26% of patients have suffered cardiac arrest before canulation.^1^Moreover, recently published trials testing VA-ECMO for CS included only patients with MI,^7^while in our cohort MI represents only 37% of cases.^1^The ECMO-CS trial, which included patients with different CS etiologies, tested mainly the time of implementation – immediate VA-ECMO and the no early (conservative) VA-ECMO. In the conservative group, 39% required downstream VA-ECMO support, which may have diluted the VA-ECMO benefit that would emerge from another irrevocably conservative approach, in the absence of inclusion of VA-ECMO to the health system.^22^

Therefore, our study highlights that, if proven effective in further studies, VA-ECMO could emerge as a cost-effective therapeutic option within the framework of the Brazilian Unified Health System (SUS). However, it is essential to acknowledge that social inequality is a hallmark of middle-income countries like Brazil, and equity remains a pressing concern.^23^Additionally, maintaining resilience in ICUs often requires a better understanding of which patients will truly benefit from intensive therapies.^24^Thus, before widespread implementation of a high-cost therapy, rigorous clinical trials encompassing a more diverse profile of CS patients and a lower incidence of cardiac arrest preceding device cannulation are needed to elucidate the role of each disease, beyond IM, in outcomes, and assess the relationship between the stages of CS and benefit of VA-ECMO.^25^In addition, the said trial differs from our cohort, where the majority of patients had SCAI (the Society for Cardiovascular Angiography and Interventions) D (contrary to studies with a predominance of SCAI C or E).^1 , 25^Further studies are needed not only to reduce uncertainties regarding the cost-effectiveness of this therapy but also to guide clinicians, and to ensure equitable access to cutting-edge medical interventions within the healthcare system.

Given the enhanced efficacy of VA-ECMO, additional challenges in its integration into the Brazilian healthcare system, from the policymakers’ perspective, include understanding the overall budgetary implications of implementation, establishing suitably equipped and trained centers for optimal device utilization, and recognizing the presence of a learning curve effect.^1^This underscores the importance of establishing specialized centers in each region, considering regional disparities and expertise, as the optimal pathway for ensuring the effective implementation of the technology.

## Conclusion

In summary, our cost-utility analysis conducted within the Brazilian Unified Health System context suggests that adding VA-ECMO to standard care may offer a cost-effective treatment option for adult patients with refractory CS, irrespective of its cause. However, the scarcity of robust efficacy data and recent randomized trials addressing specific patient subsets highlight the need for further research. Rigorous clinical trials, including a more diverse patient profile and lower incidence of immediate cardiac arrest, are essential to confirm the cost-effectiveness of VA-ECMO and ensure equitable access to advanced medical interventions within the healthcare system, especially in countries like Brazil with diverse patient populations.
